# Prepectoral Prosthetic Breast Reconstruction Without ADM Using a Subfascial Approach

**DOI:** 10.1007/s00266-024-04009-x

**Published:** 2024-05-08

**Authors:** Donald A. Hudson

**Affiliations:** UCT Private Academic Hospital, Anzio Rd, Observatory, Cape Town, South Africa

**Keywords:** Prosthetic prepectoral breast reconstruction, Fascia, No ADM

## Abstract

**Background:**

Immediate prosthetic reconstruction has evolved to a prepectoral position. A technique is described where the pectoral and serratus fascia is raised from superiorly. Initially, Vicryl mesh was used to close the superior fascial defect, but later abandoned by using primary closure for tissue expanders, or creating a pocket in the infraclavicular pectoralis muscle after prosthesis (DTI) insertion. The inframammary fold is also reinforced. Patients with a BMI > 30 have axillary liposuction.

**Method:**

Retrospective analysis over a 4-year period. Data included age, number of breasts having expanders or DTI. Prosthetic extrusion and follow-up were recorded. The percentage coverage by fascia was calculated.

**Results:**

Forty-seven patients (80 breasts) had mean age of 42 years (range 32–62), twelve patients (19 breasts) had Vicryl mesh inserted, while 35 patients (61 breasts) had closure as noted above. Tissue expanders were inserted in 39 breasts (10 mesh, 29 without). DTI (direct to implant) performed in 41 breasts (32 no mesh, 9 with mesh). Three patients with mesh developed recalcitrant seromas. The mean size of prosthesis used was 353ml (range 200–500 ml). Extrusion occurred in eight breasts (two with mesh, six without). Mean coverage of the prosthesis by fascia was 74% (range 50–100%), and nine patients also had bilateral axillary liposuction of the axillary roll. Mean follow-up was 13 months.

**Conclusion:**

Another technique for immediate prosthetic reconstruction providing an additional layer of prosthetic cover in prepectoral plane, without mesh. Applicable for all grades of ptosis. Extrusion rate is low.

**Level of Evidence IV:**

This journal requires that authors assign a level of evidence to each article. For a full description of these Evidence-Based Medicine ratings, please refer to the Table of Contents or the online Instructions to Authors www.springer.com/00266.

**Supplementary Information:**

The online version contains supplementary material available at 10.1007/s00266-024-04009-x.

## Introduction

Immediate prosthetic breast reconstruction is the commonest method of breast reconstruction [1, 2]. Historically, the prosthesis was inserted in a totally submuscle pocket [1–5], but this yielded poor aesthetic results. The development of the dual-plane for breast augmentation led to the partial submuscular plane becoming the plane of choice [1, 2]. This same plane was later used for reconstruction. However, this meant that the lower pole of the prosthesis was only covered by the potentially tenuous mastectomy skin flap—which saw the advent of ADM to provide a second layer of closure [1–8] for the prosthesis.

Additional problems of the dual-plane approach was not only animation, but also that the pectoralis muscle applied superior and lateral traction to the mastectomy flaps, distorting breast shape and compromising the aesthetic result [1, 2, 8]. Also, pectoralis muscle function was reduced [9]. An ADM not only provided an additional layer of closure, but also allowed anchoring of the IMF, thereby improving the cosmetic results. However, ADM insertion was not without complications—including infection, seroma, etc., and also increased procedural costs [1–8].

While animation was reduced with a type 1 dual-plane approach [9], it still occurred. In addition, muscle mobilisation was associated with more pain [10]. The tenting of the prosthesis over the pectoral muscle impaired the aesthetic result particularly in patients having unilateral reconstruction. Consequently, the natural progression was to insert the prosthesis in the subglandular or prepectoral plane, i.e. place where breast tissue had actually been removed [1–6]. In most cases, it appears the prosthesis was covered by a mesh of some sort [1–6]. In some cases, an absorbable mesh was used [7, 10, 11]. Recent studies have suggested that the complications of reconstruction are the same whether mesh is used or not [4, 12].

The article discusses the role of the subfascial plane, but the present study focuses on raising the combined pectoral and serratus fascia sheet from superiorly (starting infraclavicularly), and other innovations in immediate prosthetic breast reconstruction over a 4-year study period are reported. In the initial part of the study, Vicryl mesh [12, 13, 14] was used to close the superior defect (where the fascia had been transected) but analysis of the data showed no benefit, and this practice was abandoned in favour of autologous tissue closure (either primary fascial closure or, raising a small infraclavicular pectoralis flap).

## Methods

Retrospective study at a tertiary academic hospital over a 4-year period (2019–2022).

All patients underwent skin sparing or nipple sparing mastectomy. Depending on the stage of disease and risk factors, either a prosthesis (direct to implant, DTI) or tissue expander (TE) was inserted, where possible DTI is performed; however, in the case of risk factors, e.g. mild smoker (< 5 cigarettes per day) or raised BMI, etc., a tissue expander was inserted. These were clinical decisions and individualised for each patient.

Inclusion criteria: Patients with *T*1 and *T*2 tumours were > 5 mm from the pectoral fascia. All the patients in this study had proven cancer and also underwent either sentinel node biopsy or axillary dissection, as clinically indicated.

Exclusion criteria: Patients where the tumour was either invading or less than 5 mm from the pectoral fascia. Also patients with advanced local disease, those who had prior radiotherapy, were diabetic or who smoked > 5 cigarettes per day were also excluded. Patients with BMI > 36 were also excluded.

## Technique

All patients had tumescent fluid infiltration after anaesthetic induction, but before cleaning and prepping.

If the patient has a raised BMI (> 30), bilateral axillary liposuction was performed first, after tumescent infiltration to the axilla.

A skin sparing or nipple sparing mastectomy is performed by the oncological surgeon. Unless the tumour was < 5 mm to, or invading the pectoralis and/or underlying muscle, all the pectoral and serratus fascia was preserved.

In the case of grade I and II breast ptosis, a lateral oblique incision starting at the lateral areola is used for the mastectomy. In patients with grade III ptosis, a Wise keyhole skin pattern is used, in which case the deepithelialized inferior flap provides an additional layer of cover to the prosthesis or expander.

**Fig. 1:**
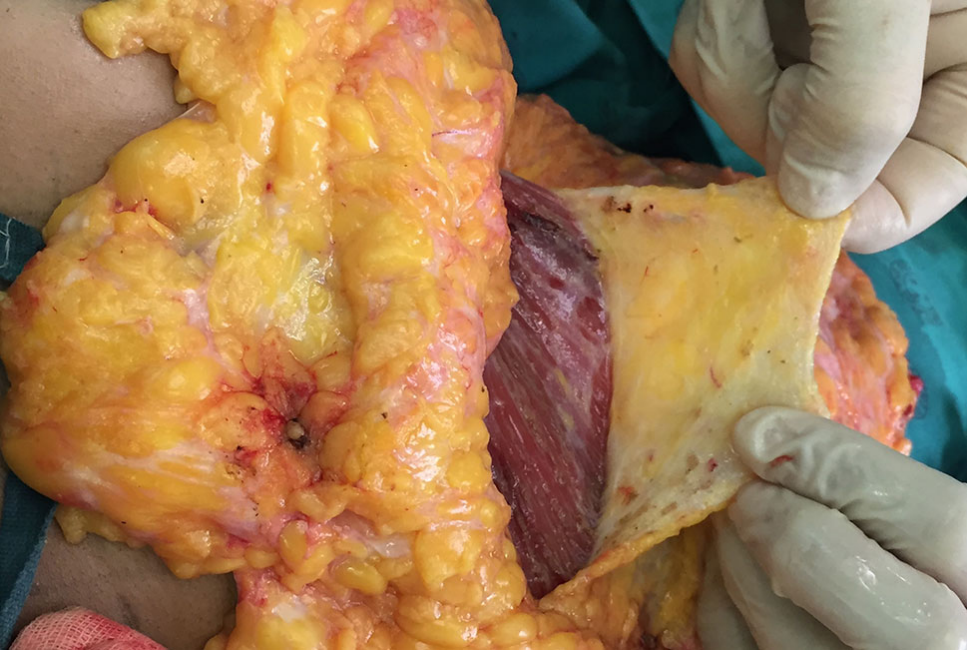
Robust fascial layer being raised from superiorly, starting inferior to the clavicle. This patient had a Wise keyhole skin pattern used for the mastectomy

Once the mastectomy has been completed, the subfascial plane is infiltrated with tumescent fluid between 150 and 200 ml per side injected using a spinal needle (see video 1). This is an important manoeuvre to enhance subfascial dissection (Fig. 1).

An incision is made from medial to lateral in the superior aspect (infraclavicular) of the pectoralis fascia, and a subfascial plane is dissected starting from above (video 2) and passes downwards (inferiorly) to the level of inframammary fold. In the lateral aspect of the breast, this subpectoral plane is extended beyond the lateral border of pectoralis major to include the fascia of serratus anterior as a single sheet.

The IMF is reinforced with a double armed 1/0 maxon suture. The IMF is reinforced centrally, i.e. in the midline of the breast. Then, it is also reinforced medially (between the central suture and the medial border of the IMF), and laterally, using 2–4 sutures. These sutures pass from the subdermis of the IMF to the rib periosteum, as a horizontal mattress suture. All the IMF sutures are inserted before each suture is tied individually.

**Fig. 2:**
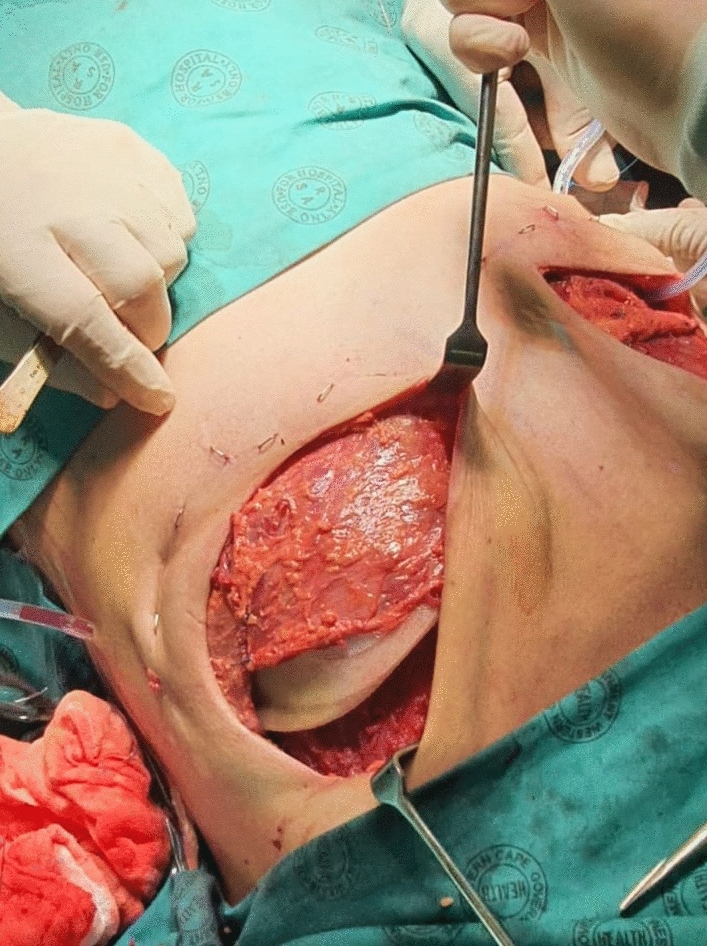
Tissue expander inserted in subfascial plane. The superior pole of the expander is visible. Photograph taken from above the patient

**Fig. 3:**
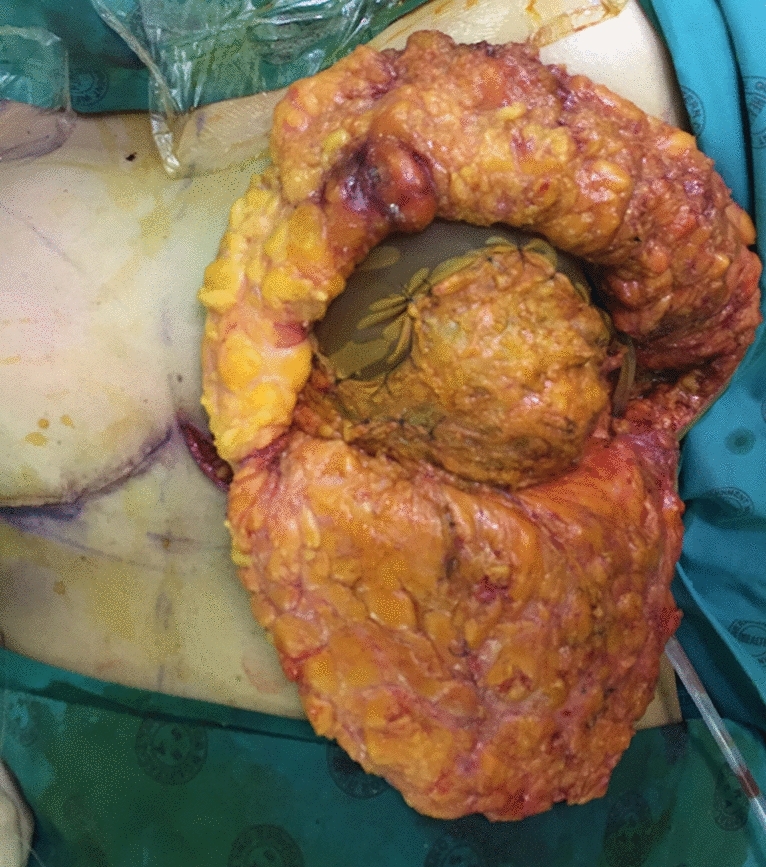
Prosthesis covered by Vicryl mesh. The photograph shows the Vicryl mesh situated superiorly, and stitched to each edge of the pectoral fascia with a prosthesis in situ. This patient had a Wise keyhole skin pattern used for the mastectomy

Once this has been completed the tissue expander (Fig. 2) or prosthesis is inserted (DTI) (Fig. 3).

Then, the defect created by opening the fascia superiorly is closed (see below).

Initially in this study, Vicryl mesh was used to close this defect (Fig. 3) after raising the pectoralis fascia from superiorly. However, a high incidence of seroma was noted. Thereafter, if a tissue expander was inserted, the fascial defect was closed primarily (video 3).

**Fig. 4:**
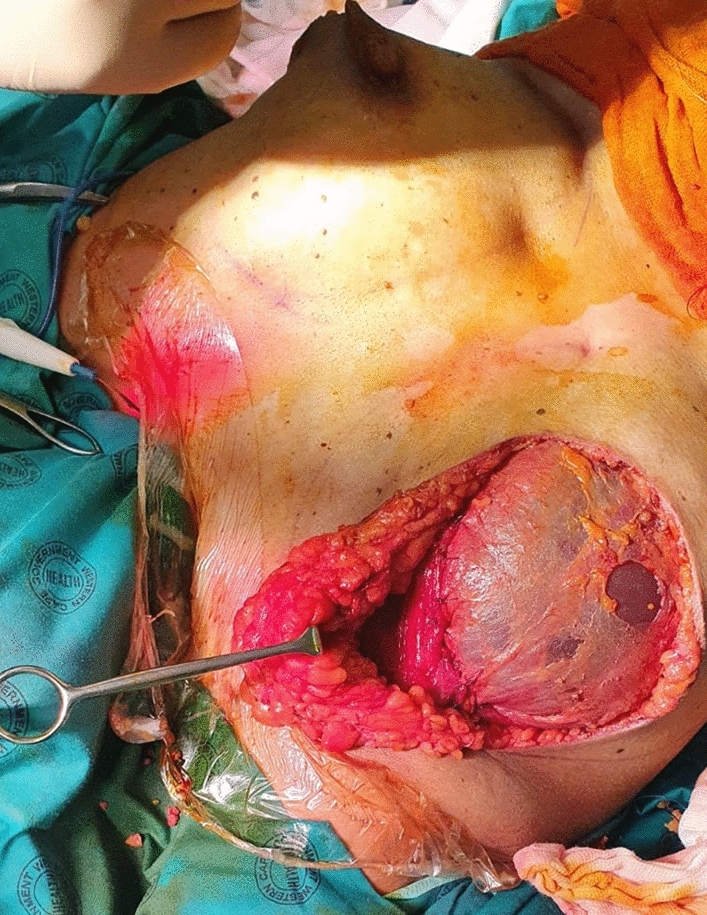
Patient having DTI, where a superiorly based pectoralis muscle pocket was created; then the pectoralis muscle was sutured to the fascia. Note hole in fascia. See also video 4

However, when a prosthesis was used (DTI), a small submuscular pocket was created in the infraclavicular aspect of the pectoralis major, which was then sutured to the fascial incision to effect closure of the pocket (Figs. 4 and video 4).

**Fig. 5:**
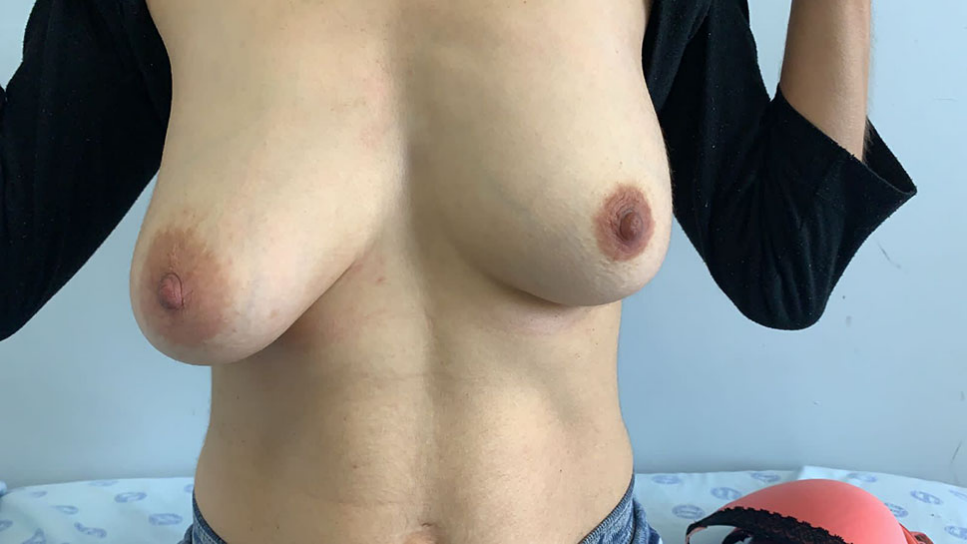
Preop photo of patient having bilateral DTI using subfascial plane. The right side had grade iii ptosis and a Wise keyhole pattern was used for the nipple sparing mastectomy. The left side had a lateral incision beginning at the areola  used for the nipple sparing mastectomy

**Fig. 6:**
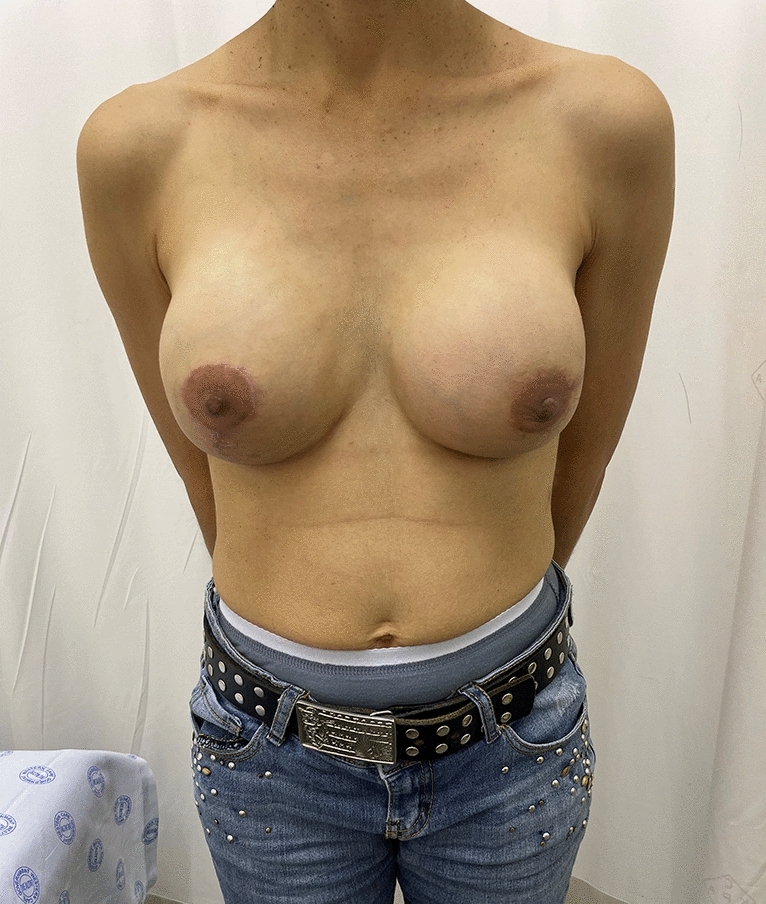
Patient in figure 5, now 8 months post op nipple sparing mastectomy and DTI

**Fig. 7:**
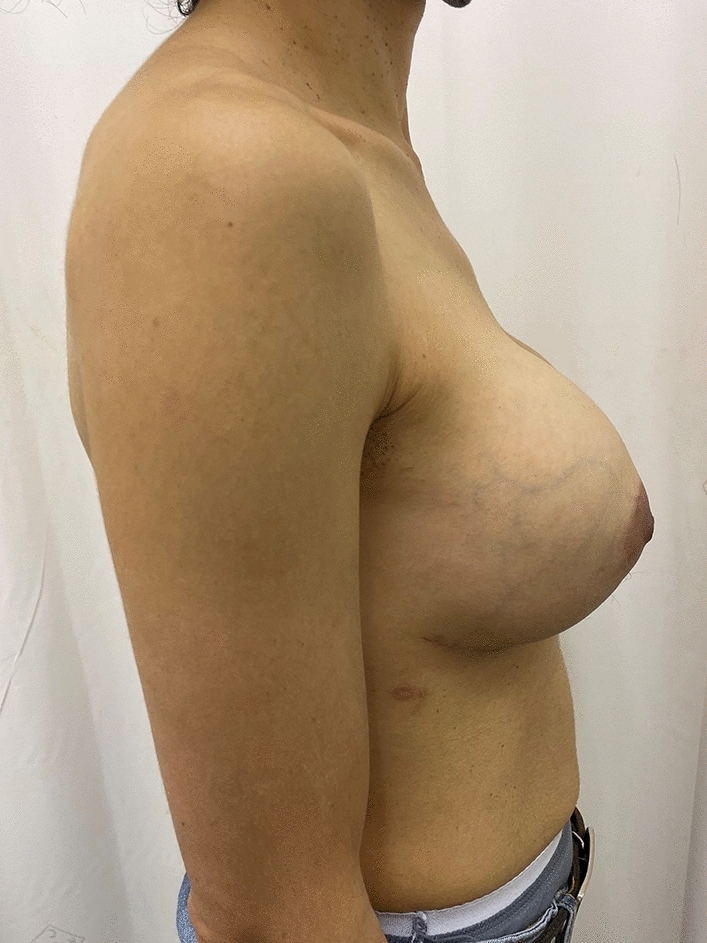
Lateral post operative view of patient in figure 5

In this study, only round, textured implants were used for direct to implant reconstruction (DTI) (Figs. 4, 5, 6, 7. 9, 10 and 11). In the patients where tissue expanders were used, these were all anatomically shaped tissue expanders. (Fig. 2) When a tissue expander was inserted, it were filled to 20% of its volume.

**Fig. 8:**
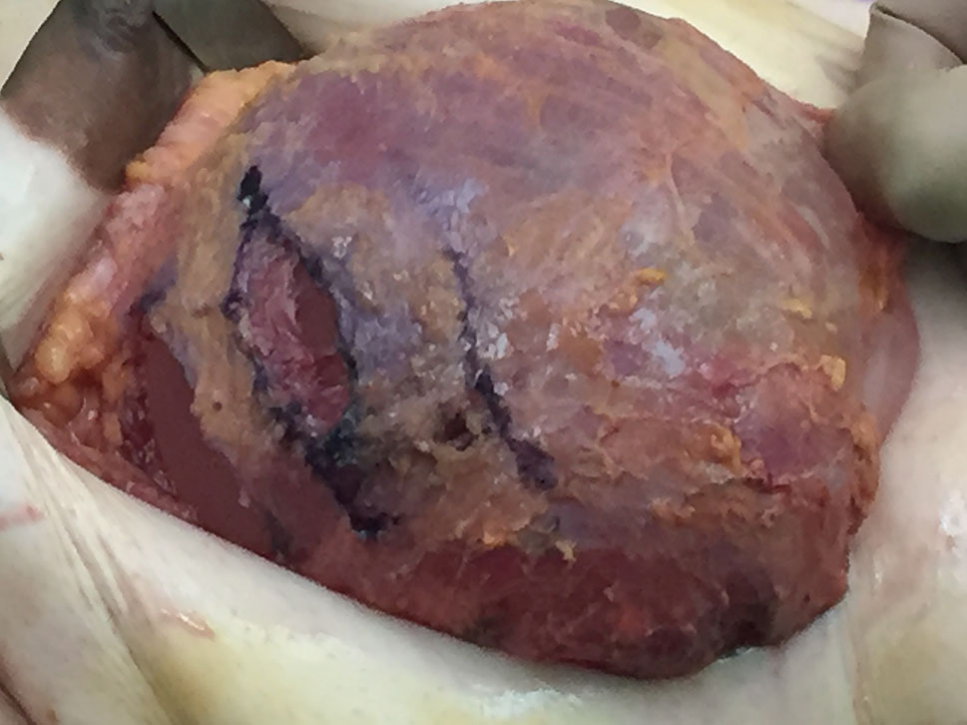
Scoring of fascia in a patient with grade ii ptosis, which allows the prosthesis prosthesis to sit more inferiorly, and fill the skin envelope. This technique can also be used to fill a bigger prosthesis. The scoring is mainly in the inferior aspect of the fascia

**Fig. 9:**
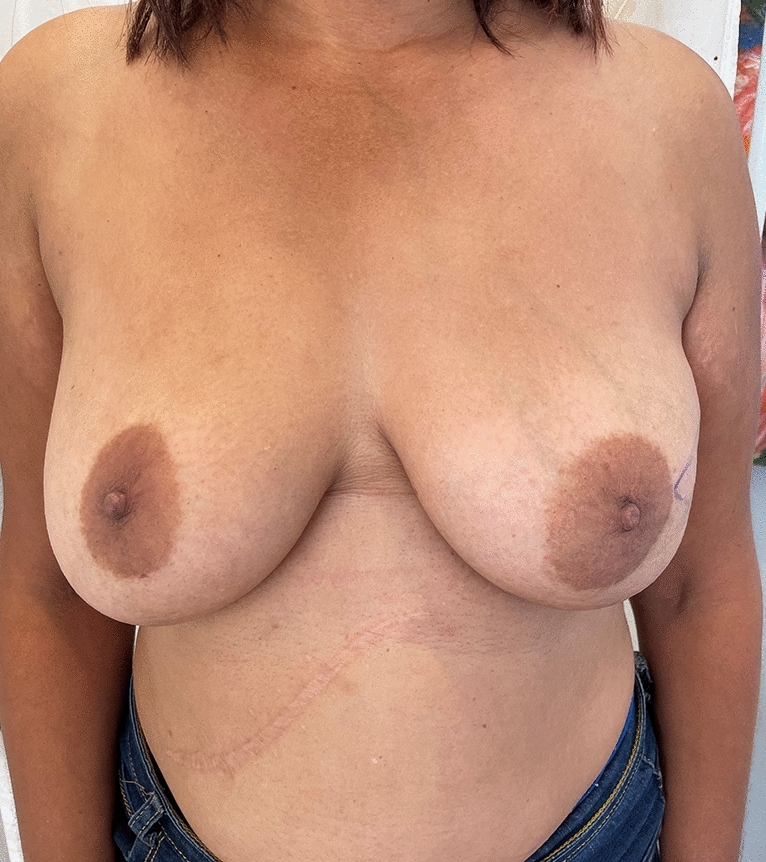
Patient who presented with left breast cancer and grade ii ptosis. She was planned to have a left nipple sparing mastectomy (only) and DTI. Because the tumour was situated close to the skin in the lateral aspect of the breast, a 2cm ellipse of skin was also removed

**Fig. 10:**
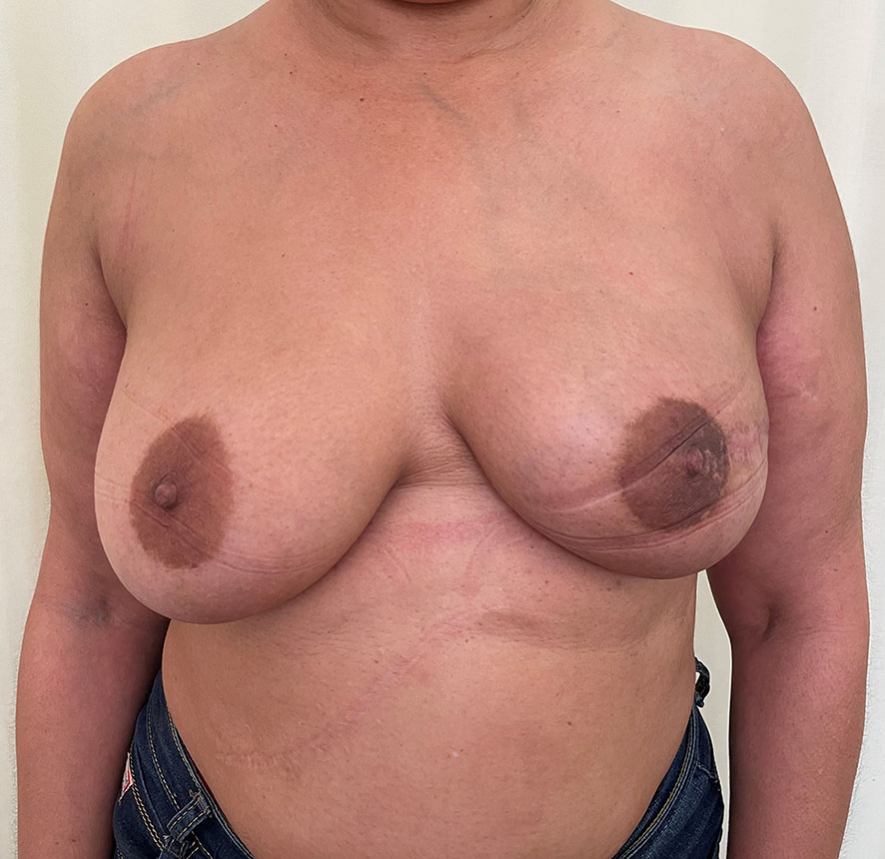
Patient in figure 9 who is now 7 months post op and had scoring of fascia and DTI

**Fig. 11:**
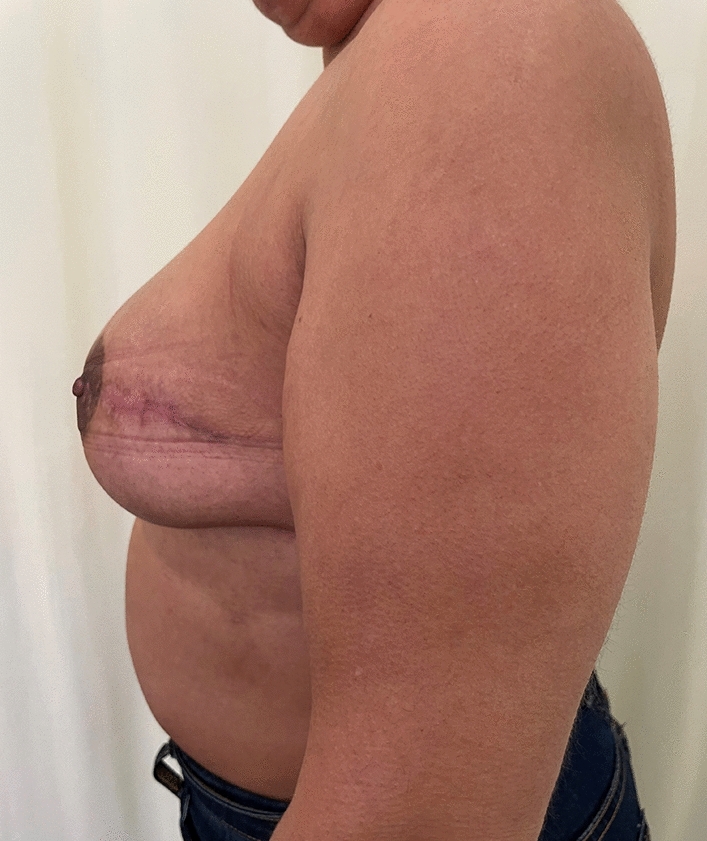
Lateral view of patient above, at 7 months post op

In patients with grade ii ptosis, the fascia is scored (Fig. 8) to allow the prosthesis to sit more inferiorly (Figs. 9, 10 and 11 and fill/expand the lower pole of the breast.

Once the expander or prosthesis had been inserted, the percentage cover of the implant (either expander or prosthesis) by the overlying fascia sheet was measured and recorded as a percentage of total facial cover (see Table 1). In some places, this fascial layer may be thin and attenuated, and in some instances, there were defects/holes in the fascia (Fig. 4) which are noted. This technique is somewhat dependant on the skill of the oncological surgeon who performs the mastectomy, as they are asked to retain the pectoralis fascia, if oncologically feasible.

**Table 1:** Patient data

	Patient numbers	Number of breasts	Mean age (yrs)	DTI in breasts	TE in breasts	Mean % of fascia cover	Liposuction: no. of breasts	Explant; no. of breasts	Mean f/u months
No mesh used	35	61	41	32	29	74%	14	6	12
Vicryl mesh used	12	19	44	9	10	70%	4	2	16
Total	47	80	42	41	39	73%	18	8	13

*Liposuction of the axilla* Note that only the lateral axillary roll, which is present in patients with a raised BMI, was performed via a stab incision beyond the lateral aspect of the breast. Most commonly, a wise keyhole pattern was used in these patients, in which case the incision was at the apex of the lateral incision of the keyhole/inverted *T*.

The study was approved by the local university ethics committee (HREC).

All procedures performed in studies involving human participants were in accordance with the ethical standards of the institutional and/or national research committee and with the 1964 Helsinki declaration and its later amendments or comparable ethical standards.

## Results (Table 1)

There were 47 patients (80 breasts), with a mean age of 42 years (range 32–62).

Twelve patients (19 breasts) has Vicryl mesh inserted, while 35 patients (61 breasts) had either primary fascial closure (after tissue expander insertion) or a small muscle flap (after DTI) raised superiorly.

Tissue expanders were inserted in 39 breasts (10 Vicryl mesh, 29 no mesh used). DTI (direct to implant) was performed in 41 breasts (32 no mesh, nine with Vicryl mesh).

The mean size of the prosthesis used was 353 ml (range 200–500 ml).

The mean % cover of the prosthesis or expander by the overlying fascia was 74 % (range 30–100%). Twelve patients achieved 100% fascial cover.

The mean follow-up of the whole group was 13 months (range 2–30 months).

Extrusion of the prosthesis or tissue expander occurred in nine patients in total. In eight of these patients, this occurred within 3 months of the mastectomy. One patient had delayed extrusion after completion of post-op radiotherapy

Nine patients with a BMI > 30 and underwent liposuction of only the lateral axillary roll. The average fat volume harvested was 90 ml (range 60–200 ml).

Patient details and results in this study who had Vicryl mesh inserted (Table 1):

Twelve patients had Vicryl mesh inserted in the superior aspect of the breast (Fig. 3). These patients had a mean age of 44 years (Range 33–59).

Two patients had grade iii ptosis and underwent a wise keyhole pattern for their mastectomy.

One patient had grade ii ptosis with fascial scoring performed after raising the fascia.

The mean coverage by the overlying fascia was 70% (range 30–100%).

Of these 12 patients, seven underwent bilateral procedures and five were unilateral (19 breasts). Tissue expanders were inserted were inserted in ten breasts, and DTI was performed in nine breasts. The average size of the prosthesis (DTI) was 350 ml (range 200–500 ml).

The mean follow-up was 16 months (range 4–26 months).

Patient results and details where prosthetic reconstruction was performed without Vicryl mesh (see Table 1):

The mean age of these patients was 41 years (range 32–62 years).

Twenty-six of these patients had bilateral reconstructive procedures performed. Figures 5, 6, 7 and Figs. 9, 10 and 11 shows a patient after bilateral nipple sparing mastectomy and DTI.

The mean size of the prosthesis used was 355 ml (range 275–500 ml).

In two patients with grade ii ptosis, the fascia was scored (Figs. 9, 10 and 11).

The mean coverage by the overlying fascia was 76% (range 40–100%).

Eight patients with grade III ptosis had a keyhole pattern used for the mastectomy.

The mean follow-up was 12 months (range 2–30 months).

## Complications

### Seroma: (A) Vicryl Mesh Patients

Three patients in the mesh group developed seroma’s that required multiple episodes of aspiration.

Once the technique to close the superior fascial defect was established (see technique above), Vicryl mesh was no longer required, and this method was then abandoned.

### Seroma: (B) Where No Vicryl Mesh Was Used

Three patients developed seromas which responded to serial aspiration.

## Extrusion

Extrusion occurred in two of 12 patients (2/19 breasts, 11%) who had Vicryl mesh inserted.

Extrusion occurred in six of 35 patients (6/61 breasts 9.8%) where Vicryl mesh was not used.

Another patient developed late extrusion after completion of post-operative radiotherapy.

## Discussion

Prosthetic breast reconstruction is the commonest method of immediate breast reconstruction. This is due to its simplicity, and also concomitant advances in mastectomy techniques, particularly nipple sparing mastectomy. Studies have shown that implant-based breast reconstruction may have a lower rate of overall complications at 1 year compared to various types of autologous reconstruction [12].

Each facet of this technique is discussed below.

### Subfascial Plane

The subfascial plane has been used in breast augmentation, with reported advantages over the subglandular plane [15]. In fact, the subfascial plane is reported to offer many of the advantages of a submuscular plane, but without the problem of animation [15]. The subfascial plane provides an additional layer of tissue cover for the prosthesis or expander in patients having reconstruction. It also prevents lateral displacement of the prosthesis/expander. The prosthesis or tissue expander was fully covered by the fascia in 12 patients in this study, i.e. there were no holes in the fascia which had been raised (Fig. 4). The mean percentage of fascial coverage achieved was 73%.

This technique is different to other approaches where a subfascial plane [16–18] is used. It begins the subfascial dissection superiorly (infraclavicularly) where the fascia is thicker [15] and thus easier to raise (video 2); tumescent infiltration greatly facilitates this process (video 1). After the expander or prosthesis is inserted, a fascial defect is created superiorly. Although the mastectomy skin flap is well vascularised at this level (infraclavicularly), it is still thin, and where a prosthesis is inserted for reconstruction, rippling may occur [19].

Initially in this study, this superior fascial defect after expander or prosthetic insertion was covered with Vicryl mesh (Fig. 3), but due to the incidence of recalcitrant seromas, this technique was abandoned. In the case of patients having anatomical tissue expanders inserted (filled to 20% of its volume), the fascia was simply closed primarily (video 3).

In the case of a direct to implant (DTI), a pocket was raised in the superior (/infraclavicular) portion of the pectoralis major muscle, enabling the upper pole of the prosthesis to be placed submuscle (Fig. 4). The inferior edge of the transected fascia is then sutured to the lower border of the pectoralis muscle flap, ensuring a contained pocket for the prosthesis (and completing a two-layered closure over the prosthesis (Fig. 4 and video 4)). This two layer closure should circumvent any rippling of the prosthesis becoming visible. A similar technique of creating an upper pectoralis pocket was described by Pitman et al [19], but they used an ADM to secure cover of the prosthesis superiorly. As this is a limited pocket in the infraclavicular portion of the pectoralis muscle, no animation was observed in this study.

The disadvantages of this plane include the limited pliability of the fascial sheet, which limits the size of the pocket which can be created. However, if a large and/ or very high profile prosthesis is required, it is possible to ‘score’ the overlying fascia to increase this capacity (Fig. 8). Fascial scoring also makes it possible to create a breast with grade II ptosis. (Figs. 10 and 11).

The disadvantage of fascial scoring is that the percentage of fascial coverage of the prosthesis is reduced. It also may be technically challenging to raise the fascia this way using only an inframammary incision to perform the mastectomy. It is also relies on the skill of the oncological surgeon retaining the pectoral and serratus fascia at mastectomy and explains why 100% fascial cover was seldom achieved (Fig. 4), and holes in the fascial flap commonly occurred. In fact, complete fascial cover was only achieved in 12 out of 47 patients.

### IMF

The definition and position of the IMF is a critical factor in breast aesthetics [20]. The IMF is attenuated by the mastectomy, irrespective of the skin pattern used. The IMF was reinforced with sutures in all patients in this study. Another advantage of lifting the fascia from superiorly and thus retaining the fascia inferiorly at the IMF is that it provides an additional layer of support at the level of the IMF. IMF reinforcement using sutures is done in two planes, i.e. a subdermal plane, and also, it is anchored to the rib periosteum not dissimilar to a previously described technique [20]. Cordero and Jazayeri [20] reported using silk sutures in their two-stage breast reconstruction. At the second stage, after removal of the expander and insertion of the prosthesis, they used a silk suture which passed from the breast capsule (not subperiosteal) to a deep dermal plane.

It is our belief that reinforcement of the IMF is mandatory whether a one-stage DTI or a two-stage tissue expander reconstruction is undertaken. In patients having DTI, it not only impedes inferior migration of the prosthesis, but importantly produces a crisp, well-defined IMF which enhances the cosmetic result. Others have also reported IMF reinforcement after both prosthetic [5] and autologous reconstruction [21].

### Liposuction

Liposuction is now commonly used to enhance the result of breast reduction [22]. The definition of the lateral aspect of the IMF may be blunted, and the axilla is ‘full’ especially in patients with a raised BMI. Liposuction is thus used to reduce the lateral breast roll particularly in patients with a raised BMI.

All the patients in this study (except one), who had axillary liposuction performed, had a wise keyhole skin pattern used, and the access incision for liposuction was at the lateral angle of keyhole thereby ensuring that only the axillary lateral roll underwent liposuction. An axillary roll post-surgery is distressing to the patient and also impairs the cosmetic result. This adds about 20 minutes to the procedure. There were no complications in this study directly related to the performance of liposuction.

**Fig. 12:**
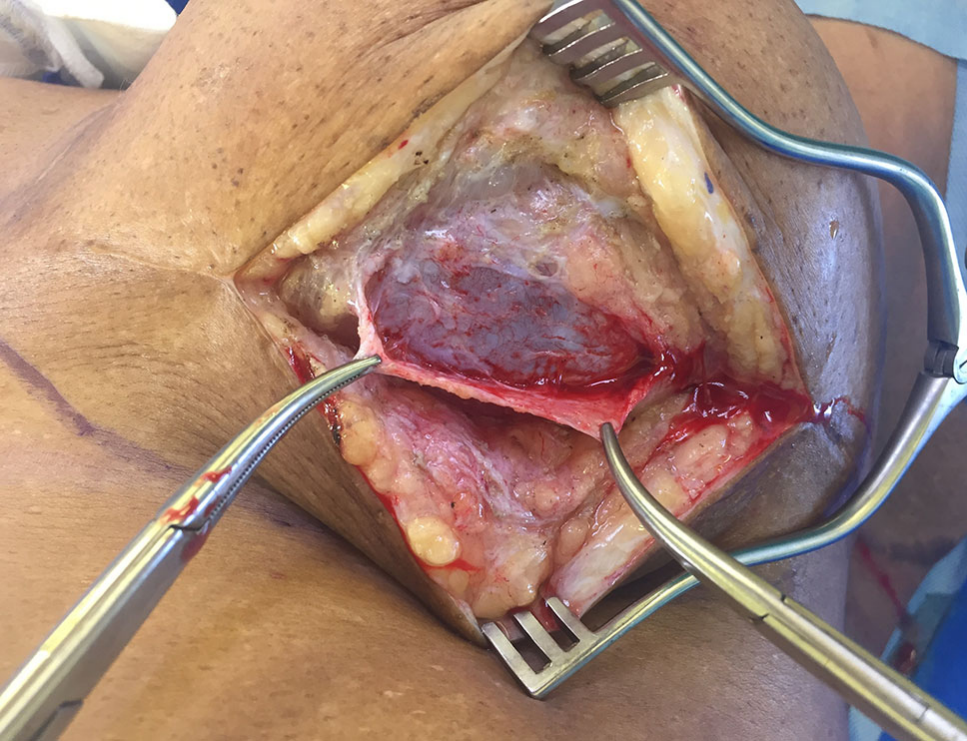
Fascial layer noted to be present at the second stage of reconstruction, in a patient having tissue expander removal and insertion of a prosthesis

There are of course disadvantages to this technique. The fascia is a relatively rigid layer, and even with scoring, there is a limit to its distensibility and the space that can be created. Hence, this technique may not be applicable if a large, high profile prosthesis is used. The biggest prosthesis used in this study was 500 ml, but the mean size used was only 353 ml. The vascularity of the described fascial flap has not been investigated but was noted to be present at the second-stage operation in patients who had had expanders inserted (Fig. 12).

There are some shortcomings in this study. The extrusion rate is 10% which is comparable to other studies [23]. Long term follow-up will be required to assess capsular contracture and the continued benefit of IMF fixation. Additionally, the incidence of rippling of the prosthesis was not investigated. Of course, fat grafting is an option to mitigate this, should it occur.

This study describes a new method to implement subfascial prosthetic breast reconstruction. The subfascial pocket provides an additional layer of autologous tissue coverage to the prosthesis (DTI) or tissue expander, and obviates the need for mesh. It can be applied to all patients with all grades of breast ptosis undergoing either skin sparing or nipple sparing mastectomy. IMF reinforcement is another component of the technique designed to enhance the aesthetic result. Patients with a high BMI will also benefit from liposuction of the lateral axillary roll.

## Supplementary Information

Below is the link to the electronic supplementary material.

Video 1 Tumescent injection of pectoral and serratus fascia. A spinal needle is used for infiltration (MP4 10757 KB)

Video 2 Raising the fascia from superiorly (starting infra clavicularly) after tumescent infiltration (MP4 10026 KB)

Video 3 After tissue expander insertion. The incision in the superior aspect of the fascia is closed primarily. The video is taken from superior (above) to the patient. Note that a bite of the pectoralis major muscle is also included in the stitch to prevent superior migration of the tissue expander (MP4 4987 KB)

Video 4 Closure of fascia using a small subpectoral pocket superiorly after DTI. The small subpectoralis pocket is created in the superior aspect of the pectoralis muscle. This muscle flap is then sutured to the pectoral/serratus fascial flap to ensure the prosthesis has an additional layer of tissue cover prior to skin closure (MOV 23347 KB)
